# Placental site trophoblastic tumour: a rare but potentially curable cancer

**DOI:** 10.1054/bjoc.1999.1061

**Published:** 2000-02-21

**Authors:** A M Gillespie, D Liyim, J R Goepel, R E Coleman, B W Hancock

**Affiliations:** Yorkshire Cancer Research Department of Clinical Oncology, Weston Park Hospital, Sheffield, S10 2SJ, UK; Department of Histopathology, Royal Hallamshire Hospital, Sheffield, S10 2JF, UK

**Keywords:** placental site trophoblastic tumour, curable, chemosensitive, surgery

## Abstract

Placental site trophoblastic tumour (PSTT) is a rare form of gestational trophoblastic disease (GTD). We have conducted an analysis of all cases of PSTT managed at the Trophoblastic Disease Screening and Treatment Centre, Sheffield, from 1984 to 1996. During this time we received 4988 registrations for GTD and managed seven cases of PSTT. A large range of interval between antecedent pregnancy and presentation was observed – the most common presenting symptoms being irregular vaginal bleeding with or without preceding amenorrhoea. Three out of seven patients had disease confined to the uterus at diagnosis and were successfully treated by hysterectomy alone. Two out of seven patients had pulmonary metastases in addition to uterine tumour and were treated with combination chemotherapy – both are alive and well. Of the remaining two patients one had distant and the other loco-regional metastases and both died despite numerous therapeutic interventions. © 2000 Cancer Research Campaign


*British Journal of Cancer* (2000) **82**(6), 1186–1190
© 2000 Cancer Research Campaign
Article no. bjoc.1999.1061, available online at http://www.idealibrary.com on IDEAL®

### Placental site trophoblastic tumour: a rare but potentially curable cancer

### Clinical experience at the Trophoblastic Disease Screening and Treatment Centre, Sheffield, UK

**AM Gillespie[1], D Liyim[1], JR Goepel[2], RE Coleman[1] and BW Hancock[1]**

[1]Yorkshire Cancer Research Department of Clinical Oncology, Weston Park Hospital, Sheffield, S10 2SJ, UK; [2]Department of Histopathology, Royal Hallamshire Hospital, Sheffield, S10 2JF, UK

**Summary** Placental site trophoblastic tumour (PSTT) is a rare form of gestational trophoblastic disease (GTD). We have conducted an analysis of all cases of PSTT managed at the Trophoblastic Disease Screening and Treatment Centre, Sheffield, from 1984 to 1996. During this time we received 4988 registrations for GTD and managed seven cases of PSTT. A large range of interval between antecedent pregnancy and presentation was observed – the most common presenting symptoms being irregular vaginal bleeding with or without preceding amenorrhoea. Three out of seven patients had disease confined to the uterus at diagnosis and were successfully treated by hysterectomy alone. Two out of seven patients had pulmonary metastases in addition to uterine tumour and were treated with combination chemotherapy – both are alive and well. Of the remaining two patients one had distant and the other loco-regional metastases and both died despite numerous therapeutic interventions. © 2000 Cancer Research Campaign

**Keywords:** placental site trophoblastic tumour; curable; chemosensitive; surgery

Gestational trophoblastic diseases (GTD) comprise a range of interrelated tumours including partial and complete hydatidiform moles, and choriocarcinoma. These tumours all arise from tissue of placental origin and due to their exquisite chemosensitivity are curable even in the presence of widespread metastases. Placental site trophoblastic tumour (PSTT) is a rare form of GTD. This clinical and pathological entity was first described in 1976 (Kurman et al, 1976) when the term 'trophoblastic pseudotumour' was used to describe a disease which followed a benign clinical course. It soon became apparent that those cases in the original report were not wholly representative of the disease spectrum and that this tumour did indeed have a malignant potential (Twiggs et al, 1981), leading to the proposal of the current nomenclature (Scully and Young, 1981). It is important to make the distinction between PSTT and other forms of GTD, as the former tumour is less chemosensitive and adverse outcomes are more common. Patients typically presented with abnormal bleeding per vaginum following a time period of amenorrhoea (Finkler, 1991; Bower et al, 1996), though a wide range of other presenting symptoms have been reported including galactorrhoea, virilization (Nagelberg and Rosen, 1995), nephrotic syndrome (Young et al, 1985; Bower et al, 1996) and polycythaemia (Brewer et al, 1992). Approximately 100 cases have been described in the English language literature and we now report our own series of seven consecutive cases.

*Received 9 June 1999*
*Revised 22 September 1999*
*Accepted 20 October 1999*

*Correspondence to:* AM Gillespie

### METHODS

We have conducted a retrospective analysis of all cases of PSTT managed at the Sheffield Trophoblastic Disease Centre from 1984 to 1996. Patients were identified from a computer database. During this time we received 4988 registrations for GTD and managed seven cases of PSTT, reflecting the rarity of this condition relative to other forms of GTD. Case records were traced and details were obtained of the clinical features and treatment received by each patient. In addition the histological features of each tumour were reviewed.

### RESULTS

The results of each case in our series is summarized in Table 1.

### Clinical features

The median age of the patients in this series was 37 years (range 26–52). The antecedent pregnancy in six cases was a full-term pregnancy; five females and one male. In the other case (patient 6) there were no previously documented pregnancies prior to the diagnosis of PSTT. In the six patients who had a previously documented pregnancy the median interval from pregnancy to diagnosis of PSTT was 24 months (range 12 months to 13 years). Irregular bleeding per vaginum and/or amenorrhoea were the presenting features in 6/7 cases; the other patient presenting in clinical shock with an acute abdomen following uterine perforation.

**Table 1** Clinical features, histological details, treatment and outcome in seven patients with PSTT

| Patient reference | 1 | 2 | 3 | 4 | 5 | 6 | 7 |
|---|---|---|---|---|---|---|---|
| Age (years) | 37 | 26 | 38 | 43 | 34 | 52 | 27 |
| Antecedent pregnancy | Female FTND | Female twins | Female FTND | Female FTND | Female FTND | No previously documented pregnancy | Male FTND |
| Antecedent pregnancy to 'presentation' (months) | 30 | 14 | 12 | 156 | 132 | – | 18 |
| Presenting symptoms | Amenorrhoea | Ruptured uterus | IBPV | IBPV | Amenorrheoa | Amenorrhoea IBPV | IBPV |
| hCG at registration (iu $l^{-1}$) | 10 | 6 | 314 | 107 600 | 2525 | 107 | 1575 |
| Number of metastases | 0 | 0 | 1 | Multiple | Multiple | 0 | Multiple |
| Sites of metastasis | – | – | Vagina | Lungs Vagina Liver CNS | Lungs | – | Lungs |
| Treatment | TAH | TAH | TAH/RT MTX Etoposide Surgery MAE MTX | D/C MAE CEC/IT MTX Cranial Irradiation | D/C MAE TAH CEC | TAH BSO | D/C D/C MTX CEC |
| Outcome | A W NED | A W NED | DOD | DOD | A W NED | A W NED | A W NED |
| Duration of follow-up (years) | 9 | 10 | 20 | 2 | 6 | 3 | 3 |
| Pathological features: | | | | | | | |
| mitoses/10 hpf (2 $mm^2$) | 5 | 2 | 15 | 14 | 1 | 1 | 5 |
| % hPL | 80 | 70 | 70 | ND | 10–20 | 90 | 10 |
| % hCG | 5 | <5 | 5 | ND | < 5 | 5 | 50 |
| Necrosis | – | – | ++ | ++ | – | + | ++ |

AW, Alive and well; BSO, Bilateral salpingo-oopherectomy; CEC, Cyclophosphamide/etoposide/cisplatin; CNS, Central nervous system; D/C, Dilatation and curettage; DOD, Died of disease; FTND, Full-term normal delivery; hCG, Human chorionic gonadotrophin; hpf, High power field; hPL, Human placental lactogen; IBPV, Irregular bleeding per vaginum; IT, Intrathecal; MAE, Methotrexate/dactinomycin/etoposide; MTX, Methotrexate; ND, Not done; NED, No evidence of disease; RT, Radiotherapy; TAH, Total abdominal hysterectomy.

### Extent of disease

At presentation, 3/7 patients had disease confined to the uterus. A further two patients had pulmonary metastases only. The other two patients had vaginal vault and widespread metastases respectively. The median beta human chorionic gonadotrophin (βhCG) at diagnosis was 314 iu $l^{-1}$ (range 6–107, 600 iu l).

### Pathology

The pathology of all cases was reviewed, though in case 4 only a single haematoxylin and eosin (H&E) section, of a metastasis to the ovary, could be traced. Hysterectomy specimens showed a poorly circumscribed tumour with variable haemorrhage or necrosis, sometimes confined to the endometrial aspect. On histological examination the tumours were characterized by gross nuclear and cytoplasmic pleomorphism of intermediate trophoblast cells. The cells infiltrated freely between muscle fibres and characteristically replaced vessel walls. Mitotic counts, necrosis and staining for hCG and hPL are shown in Table 1. The tumour in case 4 was predominantly mononuclear cells resembling atypical cytotrophoblast and intermediate trophoblast, with extensive necrosis but no biphasic pattern of mutually arranged cytotrophoblast and syncytiotrophoblast. The diagnosis is considered to be more like placental site trophoblastic tumour than choriocarcinoma. Figures 1, 2 and 3 respectively show the gross specimen, H&E stain and hPL staining in case 5, as a typical example of this tumour.

### Treatment and outcome

The three patients with disease confined to the uterus at diagnosis were all treated surgically with total abdominal hysterectomy (with ovarian conservation in 2/3 cases). None required chemotherapy and all are alive and well with no evidence of disease.

The two patients who had pulmonary metastases only are also alive and well with no evidence of disease. In neither case was the diagnosis of PSTT made prior to commencement of therapy ('low risk' in one 'high risk' in the other, see Table 2). Patient 5 developed drug-resistant disease after four cycles of 'high risk' MAE chemotherapy and underwent a hysterectomy. A histological diagnosis of PSTT was then made and the patient received a further five cycles of CEC salvage chemotherapy (see Table 2). Follow-up has been ongoing for 5 years. Patient 7 was originally treated with a working diagnosis of low-risk GTD with low-dose intramuscular (i.m.) methotrexate. However, when the results of immunohistochemical staining were known (see below) this diagnosis was changed to PSTT. CEC salvage chemotherapy was administered with a complete response (follow-up 3 years).

*© 2000 Cancer Research Campaign* 

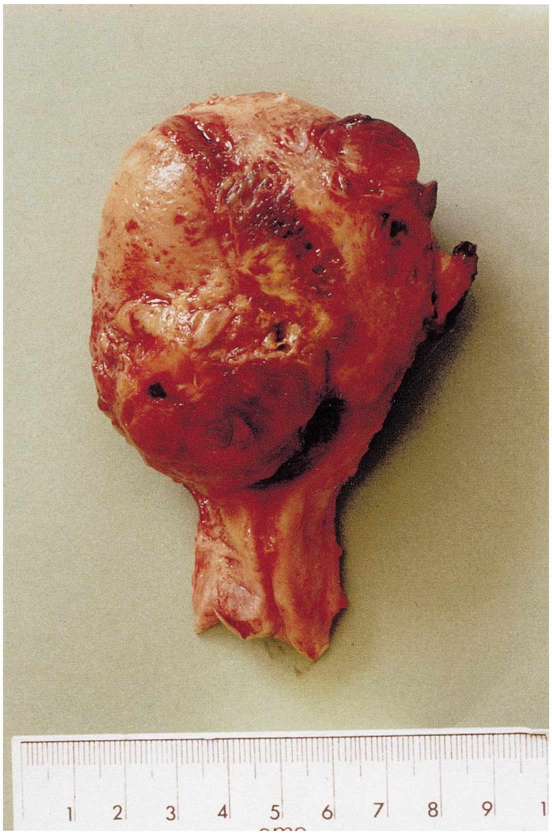

**Figure 1** Case 5 hysterectomy. Placental site trophoblastic tumour; an ill-defined mass which has replaced and permeated myometrium

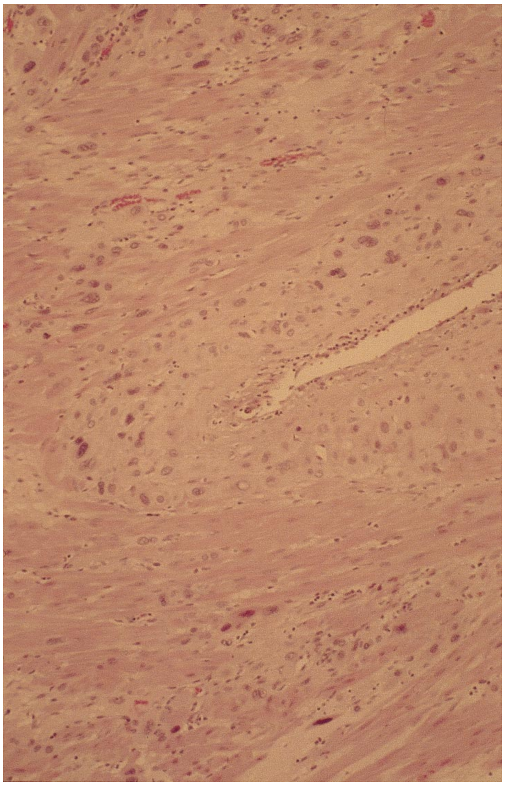

**Figure 2** Case 5. Placental site trophoblastic tumour cells with abundant pale eosinophilic cytoplasm and pleomorphic nuclei. There is invasion of myometrium and permeation of a vessel wall. H & E × 100

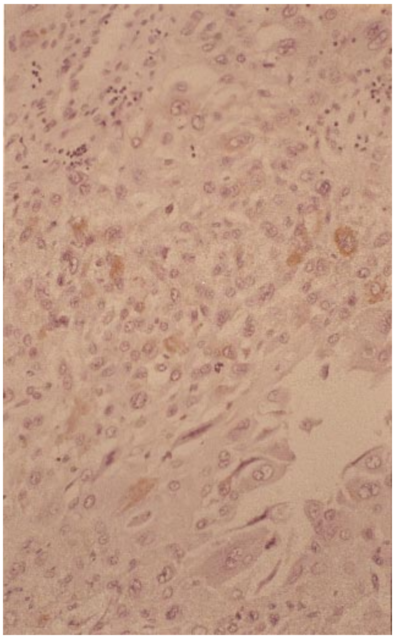

**Figure 3** Case 5. Placental site trophoblastic tumour with some of the cells positive for human placental lactogen. Immunoperoxidase anti hPL × 200

The two patients who died with PSTT both had metastatic disease at commencement of therapy at the treatment centre. Patient 3 had a particularly complicated history. Following a hysterectomy in 1968 performed for irregular vaginal bleeding a histological diagnosis of choriocarcinoma was made – though this was felt to be atypical in type. Pelvic radiotherapy was given. Sixteen years later in 1984 she presented to us with a vaginal vault recurrence, and on this occasion the histopathological diagnosis was PSTT. Retrospective histological analysis confirmed this diagnosis in the original tumour. She received four cycles of i.m. methotrexate and eight cycles of intravenous etoposide. She relapsed again in 1986 (given four cycles of MAE chemotherapy, see Table 1), in 1987 (further palliative surgery) and in 1988 (palliative methotrexate); she died of neutropenic sepsis in association with progressive disease. Patient 4 was originally considered to be a case of choriocarcinoma with widespread metastases at diagnosis. Subsequent histopathological evaluation re-classified the tumour as PSTT (see Discussion). She was initially treated with the methotrexate, dactinomycin, etoposide regimen. However, drug-resistant disease developed and salvage chemotherapy (cisplatin, etoposide, cyclophosphamide) was instituted. In addition the patient received cranial irradiation for intracerebral metastases. A complete response was never achieved and the patient died 2 years after diagnosis. Disseminated PSTT was found at autopsy.

### DISCUSSION

The establishment of National and Regional Centres for the screening and treatment of trophoblastic diseases has led to the optimization of treatment for those with most forms of GTD. However, PSTT is so uncommon that even those centres treating large numbers of women with GTD will rarely treat women with this condition. The rarity of this tumour and its varied biological behaviour means there is no consensus on optimal treatment strategies. Our series re-emphasizes many issues raised by other authors.

© 2000 Cancer Research Campaign

**Table 2** Trophoblastic disease treatment regimens at Weston Park Hospital

| | | |
|---|---|---|
| Low risk | First line 'low dose methotrexate' | Methotrexate 50 mg intramuscular alternate days × 4<br>Folinic acid 7.5 mg oral 24 h after methotrexate<br>7-day intervals between cycles |
| | Salvage 'AE' | Dactinomycin 0.5 mg day$^{-1}$ intravenous × 3<br>Etoposide 100 mg m$^{-2}$ day$^{-1}$ intravenous × 3<br>7-day intervals between cycles |
| High risk | First line 'MAE' | Arm A: Methotrexate 300 mg m$^{-2}$ intravenous<br>Folinic acid 15 mg 6-h commencing 24 h after methotrexate (eight doses, the first four intravenous)<br>Alternating with Arm B ('AE' as above) with 7-day intervals between each arm |
| | Salvage 'CEC' | Cisplatin 25 mg m$^{-2}$ intravenous daily × 3<br>Etoposide 100 mg m$^{-2}$ intravenous daily × 3<br>Cyclophosphamide 600 mg m$^{-2}$ intravenous day 1<br>7–10-day intervals between cycles |

It is known that PSTT can follow normal pregnancy, abortion or hydatidiform mole. Confirmation of the clinical impression of the ability of PSTT to arise from molar pregnancies or normal conceptions is provided by genetic analysis (Fisher et al, 1992). Some data suggest that a normal female conception is not only a common antecedent pregnancy but also an independent poor prognostic feature (Finkler, 1991); however, this is not confirmed by other investigators. In our own series a normal female conception preceded PSTT in 5/7 cases including the two deaths.

In this series the interval between antecedent pregnancy and presentation/diagnosis with PSTT was very varied (median 24 months; range 12 months to 15 years). Previous reports have suggested that a long interval from antecedent pregnancy to presentation is a significant adverse prognostic variable (Bower et al, 1996), a finding confirmed by others (Finkler et al, 1988; How et al, 1995). The common presenting feature was abnormal vaginal bleeding ± amenorrhoea; these findings are in agreement with previous reports (Finkler, 1991; Bower et al, 1996). It would be wrong to interpret this as evidence of a biologically indolent tumour – 3/7 of our cases had metastases beyond the female genital tract at diagnosis.

Histologically PSTT is a tumour with intermediate trophoblast differentiation, and a pattern of invasion similar to normal intermediate trophoblast. The poorly circumscribed mass infiltrates between muscle fibres and along vessel walls. This is in contrast to choriocarcinoma which forms a haemorrhagic mass with a characteristic biphasic histology of cytotrophoblast and syncytiotrophoblast cells in a bilaminar configuration, and intravascular growth (Silverberg and Kurman, 1991). Though it is usually possible to separate these tumours discrete categorization is not always possible. Case 4 illustrates some of the difficulties; the histology has been reviewed again by an expert gynaecological pathologist and was considered to be closer to PSTT than choriocarcinoma. The patient was managed as having PSTT, and the case is included in this series to reflect the realities of caring for patients with this rare neoplasm.

Our series conforms to the usual pattern of immunohistochemical staining of PSTT, which typically shows a high proportion of cells positive for hPL, and relatively few positive for hCG, though about 12–15% of cases have an equal or higher proportion of hCG (Kurman et al, 1984; Bower et al, 1996). Positive staining of all cell types for cytokeratins throughout the tumour is the rule, and helps in the differential diagnosis from connective tissue tumours. We also confirm the relatively poor predictive value of mitotic counts. Though our fatal cases both had a relatively high count, tumours with a low count may metastasize. Sampling variability within this tumour means that prediction of behaviour from biopsy specimens is unreliable (Hopkins et al, 1985; Finkler et al, 1998; Lathrop et al, 1988).

hCG is considered a tumour marker par excellence in evaluating treatment and follow-up of patients with choriocarcinoma and molar pregnancies – hCG value correlating with tumour bulk and persistence of disease. Unfortunately the same situation does not apply to PSTT. In our series the median hCG was 314 iu l$^{-1}$ with a range of 6–107 600 iu l$^{-1}$ with no direct correlation between tumour burden and hCG value. In fact a low hCG estimation in patients with a relatively large tumour burden should cause clinical suspicion of a PSTT. The low levels of hCG produced by PSTT reflects the cellular origin of this tumour type – intermediate trophoblast cells produce little hCG and larger quantities of hPL. In two patients (cases 6 and 7) we measured serum levels of hPL; however, the levels were barely elevated, did not serve as useful markers of disease activity and were unhelpful in guiding treatment decisions.

It is clear that the biological behavior of PSTT is variable and difficult to predict. The first diagnostic and therapeutic intervention is usually surgical. A conservative approach to surgery has been advocated by some, based on PSTT regressing after curettage (Kurman et al, 1976; Driscoll, 1984). However, this approach is probably only of value in younger women who wish to retain their childbearing potential and who understand that the tumour has unpredictable behaviour, and that the clinician's ability to successfully monitor that behaviour is currently limited. For other women with disease confined to the uterus a hysterectomy (with or without ovarian conservation, depending on risk/benefit analysis in view of the individual's age) is a more appropriate surgical intervention with a high cure rate. In our series 5/7 patients had a hysterectomy. Of the two who did not, one (case 7) wished to retain her childbearing capability and the other (case 4) had widespread metastases at diagnosis.

For women with metastases at diagnosis surgical cure is unattainable and systemic chemotherapy is required. In comparison to other forms of GTD, PSTT is less chemosensitive. Four patients in our series had metastases at diagnosis; the two patients with metastases confined to the lungs achieved remission with the use of CEC salvage chemotherapy (see Table 2). This experience is similar to other reports in the literature (Dessau et al, 1990; King et al, 1992) and contrasts with initial fears that this tumour may be unresponsive to chemotherapy (Lathrop et al, 1988).

The use of irradiation has been reported in the palliative setting with some success (Finkler et al, 1988; Wain et al, 1993; Bower

© 2000 Cancer Research Campaign et al, 1996). However, the use of irradiation without evidence of response has also been reported. In our series one patient received palliative irradiation to cranial metastases – with no evidence of tumour response.

In conclusion, PSTT is a rare tumour with unpredictable biological behaviour – nevertheless it is potentially curable. The diagnosis should be suspected when hCG levels are low relative to tumour burden. In the first instance surgery forms the cornerstone of therapy; however, contemporary practice shows that intensive multi-agent chemotherapeutic regimens can be used with some success in some of those with metastatic (particularly pulmonary) disease. Patients should be managed in Trophoblastic Disease Centres – where an experienced gynaecological pathologist should review histological diagnoses. Clinicans and patients should be aware of the major differences between this and other forms of GTD.

### ACKNOWLEDGEMENT

We are grateful to Professor Michael Wells for his expert opinion on the histopathological findings in case 4.

### REFERENCES

Bower M, Paradinas FJ, Fisher RA, Nicholson S, Rustin GJS, Begent RHJ, et al (1996) Placental site trophoblastic tumour: molecular analysis and clinical experience. *Clin Cancer Res* **2**: 897–902

Brewer CA, Adelson MD and Elder RC (1992) Erythrocytosis associated with a placental site trophoblastic tumour. *Obstet Gynecol* **79**: 846–849

Dessau R, Rustin GJS, Dent J, Paradinas FJ and Bagshawe KD (1990) Surgery and chemotherapy in the management of placental site tumour. *Gynecol Oncol* **39**: 56–59

Driscoll SG (1984) Placental site chorioma: the neoplasm of the implantation site trophoblast. *J Reprod Med* **29**: 821

Finkler NJ (1991) Placental site trophoblastic tumor: diagnosis, clinical behavior and treatment. *J Reprod Med* **36**: 27–30

Finkler NJ, Berkowitz RS, Driscoll SG, Goldstein DP and Bernstein MR (1988) Clinical experience with placental site trophoblastic tumours at the New England Trophoblastic Disease Centre. *Obstet Gynecol* **71**: 854–857

Fisher RA, Paradinas FJ, Newlands ES and Boxer GM (1992) Genetic evidence that placental site trophoblastic tumours can originate from a hydatidiform mole or a normal conceptus. *Br J Cancer* **65**: 355–358

Hopkins M, Nunez C, Murphy JR and Wertz WB (1985) Malignant placental site trophoblastic tumour. *Obstet Gynecol* **66**: 95S–100S

How J, Scurry J, Grant P, Sapountzis K, Oster A, Fortune O and Armes J (1995) Placental site trophoblastic tumour. Report of three cases and review of the literature. *Int J Gynecol Cancer* **5**: 241–249

King L, Okagaki T and Twiggs LB (1992) Resolution of pulmonary metastases with chemotherapy in a patient with placental site trophoblastic tumour. *Int J Gynecol Cancer* **2**: 328–331

Kurman RJ, Scully RE and Norris HJ (1976) Trophoblastic pseudotumour of the uterus. *Cancer* **38**: 1214–1226

Kurman RJ, Young RH, Norris HJ, Main CS, Lawrence WD and Scully RE (1984) Immunocytochemical localization of placental lactogen and chorionic gonadotrophin in the normal placenta and trophoblastic tumours, with emphasis on the intermediate trophoblast and the placental site trophoblastic tumour. *Int J Gynecol Pathol* **3**: 101–121

Lathrop JC, Lauchlan S, Ramakrishna N and Ambler M (1988) Clinical characteristics of placental site trophoblastic tumour (PSTT). *Gynecol Oncol* **31**: 32–42

Nagelberg SB and Rosen SW (1985) Clinical and laboratory investigation of a virilized woman with placental site trophoblastic tumour. *Obstet Gynecol* **65**: 527–534

Scully RE and Young RH (1981) Trophoblastic pseudotumour: a reappraisal. *Am J Surg Pathol* **5**: 75–76

Silverberg SG and Kurman RJ (1991) *Atlas of Tumour Pathology*, Third Series Fascicle 3. Tumours of the uterine corpus and gestational trophoblastic disease. Armed Forces Institute of Pathology, Washington, DC

Twiggs LB, Okagaki T, Phillips GL, Stroemer JR and Adcock LL (1981) Trophoblastic pseudotumour – evidence of malignant disease potential. *Gynecol Oncol* **12**: 238–248

Young RH, Scully RE and McCluskey RT (1985) A distinctive glomerular lesion complicating placental site trophoblastic tumour. *Hum Pathol* **16**: 35–42

Wain GV, Friedlander M, Jensen D and Truskett P (1993) Placental site trophoblastic tumour (PSTT) – an enigmatic disease: two case reports. *Int J Gynecol Cancer* **3**: 47

*© 2000 Cancer Research Campaign*
